# Barriers to Cancer Screening Uptake in Women: A Qualitative Study from Tamil Nadu, India

**DOI:** 10.31557/APJCP.2020.21.4.1081

**Published:** 2020-04

**Authors:** Selvam Mahalakshmi, Sundaram Suresh

**Affiliations:** *Department of Applied Psychology, Rajiv Gandhi National Institute for Youth Development, Sriperumpudur, Kanchipuram, Tamil Nadu, India. *

**Keywords:** Cervical cancer, breast cancer, opportunistic screening, cancer prevention, psycho oncology

## Abstract

**Background:**

The uptake for cancer screening has been consistently poor in India despite the efforts of nation-wide screening programs. Understanding the barriers and enablers among community women would aid in increasing the proportion of cancer screening uptake.

**Methods:**

Nineteen key informants including community women, service providers and a cancer survivor were interviewed using a semi-structured interview guide. Interviews were recorded and transcribed by the interviewers. Manual descriptive thematic analysis was conducted using deductive approach. Codes were given and extracted into categories which were later grouped to form themes.

**Results:**

The mean age of participants was 38 years. Among the participants, 38.9% and 16.7% underwent breast and cervical cancer screening respectively. The psychosocial factors were the major barriers for screening uptake such as fear of screening procedure and fear of being diagnosed with cancer. The other factors include lack of awareness, cultural beliefs, in addition to financial difficulties and health care system-related factors. Change in government policies to conduct mandatory screening programs, incentivization and creating awareness were reported as enablers for increasing the screening uptake among women.

**Conclusion:**

Psychosocial factors, the major barriers for screening uptake in women have remained unchanged over the years. Increasing awareness campaigns, usage of decision-making aids and changes in government policies are crucial for improving the rate of uptake and successful implementation of national screening programs.

## Introduction

In India, breast and cervix-uterus are the first and third most common sites of cancers contributing to about 144,937 cases (Globocan, 2018; Patil et al., 2019). Reports from the Tamil Nadu Cancer Registry Project (2017) observed that 56% of women were affected by cancer, out of which gynecological cancers (breast, cervix and ovary) comprised of 50%. The survival rates of breast and cervical cancers can be improved by early diagnosis (National Programme for Prevention and Control of Cancer, Diabetes, CVD and Stroke, 2017). Yet, the universal availability and accessibility of screening are debatable (Jacob, 2012), particularly in developing countries like India (Gakidou et al., 2008, Van Dyne et al., 2019, Gupta et al., 2019). While coverage is a concern, the low screening uptake by targeted women has been reported as the major challenge in cancer screening. Significant information asymmetry, economic, cultural and psychosocial factors have been identified as barriers for the low cancer screening uptake among women (Nyblade et al., 2017). 

Although the nationwide screening program (National Programme for Prevention and Control of Cancer, Diabetes, CVD and Stroke, 2017) is being implemented in India in a phased manner, not understanding and addressing the barriers will hinder the success of the program. This qualitative study attempted to explore the current barriers and enablers to breast and cervical cancer screening uptake among women in Tamil Nadu. 

## Materials and Methods

*Study design*

Descriptive qualitative study design was conducted using in-depth interviews among the selected key informants (KI). Experiences and perceptions of cancer screening were explored with particular focus on barriers to screening uptake and possible solutions.

*Study participants*

All participants in the study were purposively selected to ensure representation of various sects from the population. To obtain a triangular perspective, cancer survivor (CS), community women (CW) with and without symptoms, community service providers (SP) and professionals from oncology and community medicine were included in the study. 

*Data collection*

Interviews were conducted using a semi-structured interview guide with open-ended questions between January and March 2019 (Box 1) by a qualified psycho-oncologist (M.Phil) trained in qualitative research. Written informed consent was obtained from all participants and briefing about the cancer screening. The socio-demographic characteristics of KI was collected using a structured pro forma. The KI were inquired about barriers to cancer screening in general followed by their own experiences in screening. Among SP, their experiences about cancer screening in their routine clinical setting were explored. All the interviews were conducted face-to-face at their residence or workplace in the regional language and audio-recorded. The questions proceeded from general to specific topics to reduce interviewer and participant bias. Probing questions were asked wherever appropriate. The verbatim was transcribed on the same day by the interviewers in the same language (Tamil). The duration of interviews ranged from 20 to 30 minutes. Data collection was carried out until saturation was achieved and redundancy of information was observed. 

*Data analysis*

Two trained researchers read the transcripts to become familiar with the data and conducted the manual descriptive thematic analysis using a deductive approach to reduce researcher bias and improve interpretive credibility. The decision on coding rules and theme generation was done using standard procedures (Saldana, 2010). The contents of participants’ verbatim quotes were shortened and coded with names. The codes that covered similar ideas were merged into specific categories. Finally, the categories with similar context were grouped into themes (Creswell and Clark, 2007). Any differences between the researchers were resolved by discussion. To ensure that the results were a reflection of data, codes were related to the original data (Lincoln and Guba, 1985). Final stage of the analysis was carried out by the two researchers. The naming of categories and themes were discussed until agreement was reached. In this manuscript, the verbatim is reported in double quotations and italicized, the author explanations within quotes in square brackets and the respondents’ identities are given in round brackets and italicized. The findings were reported using ‘Consolidated Criteria for Reporting Qualitative Research (COREQ) guidelines (Tong et al., 2007).

*Ethical considerations*

The study was approved by the doctoral committee appointed by Rajiv Gandhi National Institute of Youth Development for the research degree of the first author.

## Results

*Socio-demographic characteristics of participants*

There were no refusals of consent or dropouts during participation, with a total of 19 KI, of which one KI was a male service provider (Medical Oncologist). Of the remaining 18, nine were community women, eight were service providers from health care and cancer screening setting and one was a cancer survivor. The mean age of KI was 38 years, ranging from 32 to 58 years. Only 38.9% and 16.7% of the KI underwent breast and cervical cancer screening respectively. Table 1 shows the characteristics of participants.

*Barriers and Enablers*

Table 2 shows the barriers and enablers for cancer screening reported by KI in the context of themes, categories and codes. Three broad themes for barriers and two broad themes for enablers are described below. 

*Barriers*

*Theme 1: Psychosocial and Individual Factors*

Women were found to have psychological barriers like fear, anxiety, embarrassment, shyness, negligence which were influenced by social convictions and lack of awareness.

*i. Nihilistic attitude towards cancer*

Many participants reported that fear of being diagnosed with cancer was a major barrier to screening uptake. It was also associated with fear of discrimination by family and society, stigma related to cancer (that cancer is equivalent to death) and the lack of understanding about cancer (that cancer is contagious). A few stated, 

*“I am scared, what will I do, if they say, I have cancer” (CW1, CW3,CW8,SP1)*

*“[I’m] Scared, the family members may not mingle with us casually. If the society comes to know that is all, [I] fear [that], they will isolate us” (CW6)*

*“If they say, we have the disease after the screening, our life is gone after that, the life is over, that kind of negative thoughts [prevent going]”(CW5)*

*“This is like a contagious disease, if one is diagnosed of cancer, we should not allow children near them” (CW1)*

The same was reiterated by SPs as well, *“Scared that the life will be over with that, and they will die” (SP1)*

Few women expressed concerns about the prolonged treatment and multiple visits and associated financial implications.

*“If we have to go for screening, the situation is that we need to go to hospital eight to ten days” (CW6, CW1)*

*“One day, they should spend” (SP1)*

*“Time will be waste, if we go it will take one day” (CW2)*

*“The treatment expenses [of cancer] are high” (CW5, CW6)*

*ii. Lack of awareness about cancer screening*

All the participants perceived that lack of awareness about cancer screening was a barrier for not participating in the screening program. 

*“There is no adequate amount of awareness [about cancer screening] among people” (CW4, CS1).*

Cancer screening was frequently described as a painful and uncomfortable process.

*“We cannot bear the pain” (CW1)*

*“First of all, that [mammogram] will be very painful, that is also one reason for my fear” (CW2)*

*“The fear related to the pain during the screening is also one of the important reasons” (CW8)*

Most of the women reported that being asymptomatic, feeling fit or healthy does not necessitate screening and the cause for not approaching the doctors for screening. Procrastination was also observed due to the lack of understanding of the rationale of screening. 

*“Thinking that we can do little later, maybe after 15 days” (CW1)*

*“If there are any symptoms, we can go, otherwise why should we go. The attitude is that after 40 or 45 years, the system will start functioning slow, then we can do, what is the urgency”(CW2)*

*“I don’t have any symptoms, why should I check” (SP6)*

*“I am fine only” (SP3, SP8).*

Lack of awareness also found among the family members and elders was stated as,

*“Even before, we go to the screening, they will stop us saying, you will not get such diseases, why are you imagining yourself” (CW1)*

*“A few relatives will ask, why are you going to the hospital when you don’t have any problem” (SP3)*

*iii. Negligence*

An attitude of carelessness regarding their health was seen in women as mentioned by a CW.

*“We are fine only if anything comes let us see at that time” (CW9)*

*iv. Embarrassment or shyness*

The embarrassment of revealing their body parts was a commonly perceived notion that caused hesitation in women. It was difficult for women to break their conventional mindset imbibed with cultural norms for the purpose of screening. 

*“Shyness, they need to expose the private parts to doctors (Breast and cervix)” (SP2, SP3)*

*“People are shy, that is why refusing to go” (CW3)*

v. Superstitious beliefs 

A common social stigma was identified among CW that related to the occurrence of cancer with their misdeed. They believed that going to church could cure them of their sins as well as the disease.

*“I did not commit any sin” (CW3, CS1)*

*“This disease will come only to people who commit sin” (SP8)*

*“If we go to church, it will be alright” (CS1)*

*Theme 2: Cultural and financial factors*

Cultural hindrances were found to be interlaced with familial barriers like lack of family support, household responsibilities. Financial difficulties were also reported as a major concern by the women. 

*i. Family-related barriers*

Women reported that they needed a companion as some were not habituated to going out alone. There was no support from the family to go for screening in some cases. 

*“More than 50% of the spouses do not co-operate” (CS1)*

*“Sometimes, the husband will say not to go for screening” (CW6)*

Women with household and childrearing responsibilities were not willing to give up on their duties in order to attend the screening.

*“Family ladies have so much at home, for example, taking care of children” (CW8)*

*“If they have adolescent girls, they will say they cannot leave them at home alone and come” (SP7)*

*ii. Economic barriers*

In many families women were still the bread-winners, working daily-wage jobs, and would lose a day’s wage if they attended the screening. Women felt the screening cost to be high and an additional expense.

*“[If we go to screening], we lose our daily wages” (CS1)*

*“Firstly, if we go for screening, it will cost about Rs.4000 for breast and cervical screening. Many people may think [hesitate], because they need to spend more money”(CW1)*

*Theme 3: Health care system-related factors*

*i. Lack of trust in doctors and hospitals*

Pervasive distrust on the health care facilities, both private and Government, was observed in the community. The KI mentioned that doctors from government hospitals were unavailable or unreceptive based on their previous experiences.

*“Don’t believe corporate hospitals, they will prescribe investigations unnecessarily” (CW2)*

*“At the Government hospitals, there is no regulation on the doctor’s visiting time to the hospital” (CW6)*

*“... [At government hospital], he [the doctor] said, we cannot do anything hereafter, that is all, they behaved very harsh, …” (CS1)*

*ii. Poor accessibility due to geographic location*

Women reported that screening was inaccessible to those in villages due to inadequate facilities.

*“In villages, there are no facilities at the primary health centres to do the cancer screening” (CW8)*

*iii. Male health service providers*

Screening conducted by male physician or technicians was found to be an obstacle in cancer screening. Women felt diffident and reluctant to undergo cancer screening if male health care providers conducted the screening procedures. 

*“Men are conducting the screening test [mammogram], it will be ok if women take [mammogram]. If we go to scan centrs, more than ladies, in many places men are only there, that is why women refuse to go”(CW3)*

*“We hesitate to show to male doctors” (CW4)*

*“If the doctor is sometimes male, people definitely feel shy and hesitate to show their breast to them” (CW8, CS1)*

*Enablers*

*Theme: Intensification of culture-specific IEC activities - Awareness generation*

The participants mentioned that creating awareness and educating the public about the need for screening could help reach to the community. Media was considered an essential tool to spread knowledge among people.

*“Need to conduct awareness programs, can insert handbills through newspapers, through Television…” (CW4)*

*“Can show someone who is cured of cancer as a role model” (CS1)*

*It was also suggested that regular camps should be conducted extensively.*

*“Can conduct screening camps” (CW2)*

*Theme 5: Policy changes – screen and treat, financial support*

Policy changes to increase the availability and accessibility to screening facilities, appointing female staff incentivization were suggested by the KIs.

*i. Government policies*

Participants recommended that the government should make it compulsory for women to undergo cancer screening and that women have increased access to these services.

*“The Government should bring a policy to screen all the women compulsorily” (SP1)*

*“The screening facilities should be made available in all the hospitals” (CW7, CW8)*

*“The camp should be near their place” (CW2)*

*“The facilities should be made available near their villages” (SP2)*

*ii. Financial support*

As for the financial burden, it was suggested that the screening should be done at low cost or incentives could be provided to attend cancer screening. 

*“We should advertise that the screening will be done at low cost” (CW4)*

*“Incentives can be provided. Then the people from below poverty line will come forward to do cancer screening” (SP1)*

*iii. Appointing female service providers*

Appointing more women doctors and technicians for the screening programs could help women overcome fear and embarrassment leading to a higher proportion of women attending the cancer screening. Appointing doctors from the same community will improve the level of comfort and trust in the system. 

*“We need to take them to familiar doctors, then without fear, they can do the screening” (CW5)*

*“If women do the screening/mammogram it will be good” (CW3)*

**Box 1:** Thematic Questions Used during in-Depth Interviews

Interview questions	Probing questions
Why do women hesitate to undergo cancer screening?	Do you think women feel embarrassed to attend screening programs?
	Is it due to a lack of awareness?
	Is it due to the inadequacy of hospitals to provide screening facilities?
	Do you think facilities are accessible to women near their living area?
	Do doctors and other health professionals have adequate knowledge to conduct screening?
	Do women feel scared of the screening procedures?
	Are women afraid of getting diagnosed with cancer after screening?
	Is it because they might call for multiple visits?
	Do you think the screening procedures are costly?
	Do women get husband and family support for attending cancer screening programs?
	Is it because women feel they are healthy at present?
	Is it important to get screened for cancer even if there are no symptoms?
	Are there any other reasons?
Have you ever undergone cancer screening?	If no, why?
	If yes, what motivated you to undergo cancer screening?
How to motivate the community women to undertake cancer screening?	

**Table 1.** Participant Details

Variables	N (%) (N=19)
Age in years [Mean (SD)]	38 (8.48)
Gender	
Male	1 (5.3)
Female	18 (94.7)
Category	
Survivor	1 (5.3)
Community women	9 (47.4)
Health care providers	9 (47.4)
Breast cancer screening done (n=18)	7 (38.9)
Cervical cancer screening done (n=18)	3 (16.7)

SD, Standard Deviation

**Table 2:** Perspectives of Community Women, Survivor and Health Care Providers on Barriers and Enablers for Cancer Screening

Themes	Categories	Codes
**Barriers for cancer screening**		
Psychosocial and Individual Factors	Fear of cancer diagnosis	Fear of screening outcome
		Fear of family rejection
		Stigma related to cancer
		Cancer is contagious
		Fear about treatment cost
		Prolonged treatment
	Lack of awareness about cancer screening process	Lack of awareness about screening
		Pain during screening procedures
		Misguidance from elders in the family
		Time consuming
		Absence of symptoms
		Feeling fit
		Procrastination
	Negligence	Carelessness about their health
	Embarrassment	Shyness to reveal their body parts
	Social stigma	Cancer is the consequence of sin
Cultural and financial factors	Family barriers	Lack of family support
		Lack of spousal support
		Responsibilities of women - household work/ child care
	Economic issues	High screening cost
		Loss of income
Health care system related factors	Lack of trust in hospitals and doctors	Lack of knowledge or skills about cancer screening among doctors
		Lack of trustworthiness toward private hospitals
		Unapproachable government doctors
		Non-availability of doctors in government hospitals
	Poor accessibility due to geographical location	Non-availability of screening facilities at PHCs
	Male health service providers	Male physician and male technician
**Enablers for cancer screening**		
Intensification of culture specific IEC activities - Awareness generation	Advertisements	Media, mobile, pamphlets, women movie personalities, role models, colleges, camp
	Awareness	Screening camps and awareness programs
Policy changes – screen and treat, financial support	Government policies	Make it mandatory by government
		Make it reachable
	Financial support	Reduce the screening cost
		Provide incentive
	Appointing female health service providers	Women doctors and lab technicians

## Discussion

The study qualitatively explored the barriers and enablers for screening uptake among women. Psychosocial, economic, cultural and system related factors were identified. 

The strength of this study was that, the population consisted of educated and uneducated male and female participants from rural and urban communities, professionals including oncologists, epidemiologist and other health care providers belonging to different socio-economic backgrounds representing variation in the study population. Hence the study could acquire different dimensions in its viewpoint. There were some limitations in our study. Firstly, the study population was small and there was not enough representation from each category. Secondly, as the study was conducted in a specific Indian region, generalizability is difficult.

Fear of being diagnosed or of the examination procedure were reported as barriers in previous studies (Agurto et al., 2004; Allahverdipour et al., 2011; Malhotra et al., 2016) as in the current study. Embarrassment to reveal their body parts, especially with male health service providers, not necessitating screening in asymptomatic conditions and lack of family support were also found in the current study confirming previous findings (Devarapalli et al., 2018; Marlow et al., 2015; Nyblade et al., 2017; Szalacha et al., 2017).

In previous literatures, cultural beliefs have been identified as a significant barrier (cancer as sin, the result of immorality) in women to undergo cancer screening as they prominently influence the level of understanding and knowledge about these cancers (de Cuevas et al., 2018; Gupta et al., 2015; Lee, 2015; Meana et al., 2001; Modibbo et al., 2016; Szalacha et al., 2017) and interventions addressing them have also produced results (Adunlin et al., 2019; Pratt et al., 2019). Financial concern was reported both in the current study and previous studies (Malhotra et al., 2016). Nevertheless, screening conducted at no cost for all eligible women by the current nationwide program in India might help in addressing this issue. The main enablers mentioned in our study were creating awareness, policy changes by the government including availability of facilities and incentivization. Government has been providing incentives for Tuberculosis patients to continue receiving treatment and to mothers undergoing institutional deliveries in an attempt to reduce infant and maternal mortality rate. Similar incentivization to women attending cancer screening could improve the rate of screening uptake. 

The currently prevailing barriers and enablers in the society, mentioned in this study have been consistently reported as determinants of screening uptake over the years (de Cuevas et al., 2018; Devarapalli et al., 2018; Dinshaw et al., 2007; Gupta et al., 2015; Kulkarni et al., 2019). Despite repeated researches conducted with this focus, barriers influencing cancer screening uptake have not changed for the past two decades. This might be attributed to the society’s low sensitivity about screening of women related cancers. Awareness for women cancers were reported as poor (Bora et al., 2016; Khokhar, 2009) and lack of media attention towards cancer control in India is prevalent (Gupta et al., 2015). 

Usage of decision aids will help in increasing the rate of acceptance in contemplating stage, thus improving the screening uptake. Decision aids have been shown to reduce indecisiveness, improve consensus on values and choices as well as improve knowledge (Barratt et al., 2004). IEC and screening are the two main components in cancer prevention recommended by the World Health Organization (Bora et al., 2016). The interim results of a community-based cancer screening conducted in Mumbai showed a compliance rate of 85% and 70% for breast and cervical cancer screening where the women were sensitized about the reproductive organs, cancer symptoms and early detection (Mishra et al., 2015). IEC method and targeted intervention programs have been effective to improve participation in breast and cervical cancer screenings (Jacob, 2012; Rao et al., 2005). Although decision making aids are widely used for cancer treatment, its role in cancer screening is still unexploited. In India, massive mobilization of the community, effective micro-planning and compound communication approach have been the foundation to eradicate polio in the country (Thacker et al., 2016). Such galvanizing social movement regarding women cancer screening is crucial to reach out to maximum population. Empowering through awareness and use of decision making aids are the need of the hour for mass mobilization of the society and improving the uptake of women cancer screening.

In Conclusion, This study reiterates the psychosocial barriers and enablers that have been prevalent in the community women over the period of time. It was inferred that the factors hindering the cancer screening uptake have remained unchanged for about two decades. Addressing these barriers by creating awareness, using decision making aids and changes in government policy are the means to strengthen the current NPCDCS program. 

## References

[B1] Adunlin G, Cyrus JW, Asare M, Sabik LM (2019). Barriers and facilitators to breast and cervical cancer screening among immigrants in the United States. J Immigr Minor Heal.

[B2] Agurto I, Bishop A, Sánchez G, Betancourt Z, Robles S (2004). Perceived barriers and benefits to cervical cancer screening in Latin America. Prev Med (Baltim).

[B3] Allahverdipour H, Asghari-Jafarabadi M, Emami A (2011). Breast cancer risk perception, benefits of and barriers to mammography adherence among a group of Iranian women. Women Health.

[B4] Barratt A, Trevena L, Davey HM, McCaffery K (2004). Use of decision aids to support informed choices about screening. Br Med J.

[B5] Bora K, Rajbongshi N, Mahanta LB, Sharma P, Dutta D. (2016). Assessing the awareness level of breast and cervical cancer: a cross-sectional study in northeast India. Int J Med Sci Public Heal Online.

[B7] de Cuevas RMA, Saini P, Roberts D (2018). A systematic review of barriers and enablers to South Asian women’s attendance for asymptomatic screening of breast and cervical cancers in emigrant countries. BMJ Open.

[B8] Devarapalli P, Labani S, Nagarjuna N, Panchal P, Asthana S (2018). Barriers affecting uptake of cervical cancer screening in low and middle income countries: A systematic review. Indian J Cancer.

[B9] Dinshaw K, Mishra G, Shastri S (2007). Determinants of compliance in a cluster randomised controlled trial on screening of breast and cervix cancer in Mumbai, India. Oncology.

[B10] Gakidou E, Nordhagen S, Obermeyer Z (2008). Coverage of cervical cancer screening in 57 countries: low average levels and large inequalities. PLoS Med.

[B12] Gupta A, Shridhar K, Dhillon PK (2015). A review of breast cancer awareness among women in India: Cancer literate or awareness deficit?. Eur J Cancer.

[B13] Gupta R, Gupta S, Mehrotra R, Sodhani P (2019). Risk factors of breast cancer and breast self-examination in early detection: systematic review of awareness among Indian women in community and health care professionals. J Public Heal [Epub Ahead Print].

[B14] Jacob M (2012). Information, education &amp; communication: corner stone for preventing cancer of the cervix. Indian J Med Res.

[B15] Khokhar A (2009). Level of awareness regarding breast cancer and its screening amongst Indian teachers. Asian Pac J Cancer Prev.

[B16] Kulkarni SV, Mishra GA, Dusane RR (2019). Determinants of compliance to breast cancer screening and referral in low socio-economic regions of urban India. Int J Prev Med.

[B17] Lee S-Y (2015). Cultural factors associated with breast and cervical cancer screening in Korean American women in the US: An Integrative Literature Review. Asian Nurs Res (Korean Soc Nurs Sci).

[B19] Malhotra C, Bilger M, Liu J, Finkelstein E (2016). Barriers to breast and cervical cancer screening in Singapore: a Mixed Methods Analysis. Asian Pac J Cancer Prev.

[B20] Marlow LAV, Waller J, Wardle J (2015). Barriers to cervical cancer screening among ethnic minority women: A qualitative study. J Fam Plan Reprod Heal Care.

[B21] Meana M, Bunston T, George U, Wells L, Rosser W (2001). Older immigrant Tamil women and their doctors: attitudes toward breast cancer screening. J Immigr Health.

[B22] Mishra G, Dhivar H, Gupta S, Kulkarni S, Shastri S (2015). A population-based screening program for early detection of common cancers among women in India – methodology and interim results. Indian J Cancer.

[B23] Modibbo FI, Dareng E, Bamisaye P (2016). Qualitative study of barriers to cervical cancer screening among Nigerian women. BMJ Open.

[B24] Nyblade L, Stockton M, Travasso S, Krishnan S (2017). A qualitative exploration of cervical and breast cancer stigma in Karnataka, India. BMC Womens Health.

[B25] Patil A, Salvi N, Shahina B (2019). Perspectives of primary healthcare providers on implementing cancer screening services in tribal block of Maharashtra, India. South Asian J Cancer.

[B26] Pratt R, Mohamed S, Dirie W (2019). Testing a religiously tailored intervention with Somali American Muslim Women and Somali American Imams to increase participation in breast and cervical cancer screening. J Immigr Minor Heal.

[B27] Rao RSP, Suma N, Nair NS, Kamath VG (2005). Acceptability and effectiveness of a breast health awareness programme for rural women in India. Indian J Med Sci.

[B29] Szalacha LA, Kue J, Menon U (2017). Knowledge and beliefs regarding breast and cervical cancer screening among Mexican-Heritage Latinas. Cancer Nurs.

[B31] Thacker N, Vashishtha V, Thacker D (2016). Polio Eradication in India: The Lessons Learned. Pediatrics.

[B32] Tong A, Sainsbury P, Craig J. (2007). Consolidated criteria for reporting qualitative research (COREQ): a 32-item checklist for interviews and focus groups. Int J Qual Health Care.

[B34] Van Dyne EA, Hallowell BD, Saraiya M (2019). Establishing baseline cervical cancer screening coverage- India, 2015–2016. MMWR Morb Mortal Wkly Rep.

